# Progress in Bacteriology

**Published:** 1902-09-13

**Authors:** 


### PROGRESS IN BACTERIOLOGY.

**Rabies.**—Krasmitski,[1] experimenting on rabbits, finds that a very rapid and complete immunity is conferred if, instead of using dried nervous material, the rabies virus is itself introduced intravenously. This may be done with safety, provided that the emulsion be heated to 37° C., and be filtered and diluted and slowly introduced. In persons severely bitten on exposed surfaces this rapid induction of immunity is of the greatest practical importance, and that the new procedure may be applied to human beings with impunity has been demonstrated by Wysokowicz, who has treated on these lines 70 cases, admitted to the institute at Kief, suffering from the bites of dogs with rabies. During the year 1901, 1,318 persons supposed to be suffering from rabies have been treated at the Pasteur Institute.[2] Of these five died—a mortality of ·38 per cent., which is slightly higher than that of recent years. Four of the fatal cases had been bitten by dogs certified by veterinary examination to be suffering from rabies; in the fifth fatal case there was only a suspicion that the dog was so suffering. Of 171 persons bitten by dogs, which by subsequent inoculation experiments were proved to be suffering from rabies, and who were treated at the institute, all recovered. Three persons developed rabies before their course of treatment could be completed, and all died. In addition to the institute at Paris there are five other centres in the provinces for the treatment of rabies. Stanley[3] has treated 17 cases of rabies in the Shanghai Pasteur Institute, with two deaths. In both of these cases, however, the treatment had been interrupted, and death followed soon after the bite. The cases in Shanghai are few, but are characterised by a very short incubation period. It is consequently only found necessary to inoculate a fresh rabbit every other day, and this ensures a sufficient supply of cords for the necessary intensive treatment. Intracerebral inoculation is preferable to the ordinary subdural method.

**Molluscum Contagiosum.** — White and Robey[4] review the literature of this subject. Two theories have for some time held the field, the one maintaining that the growths are of pilo sebaceous origin and the other that they are of the nature of epithelioid cancer. In 1881 Angelucci described an organism as being the exciting cause of the disease, and named it the Bacterium Lepogennum; in 1882 Neisser expressed the opinion that the organism was a variety of the gregarinidæ, and four years later specified the coccidia; other investigators have described the constant presence of micrococci. Robey squeezed out the contents of the tumors and made cultures on a variety of media including a special fluid medium made from chopped skin. The only organism found in ærobic and anærobic cultures were staphylococci, and these in no way differed from those normally found in the skin. There is no evidence to show that this condition is in any way associated with a specific organism, and from the study of a large number of sections, White concludes that the tumours are the result of an extraordinary metamorphosis of rete cells into keratin.

**Bacteria in Food Stuffs.**—Klein[5] has recently conducted an investigation to ascertain whether pathogenic microbes accidentally gaining access to cereal products can for any length of time survive in them and can thus confer on food materials, of which these cereals are ingredients, sustained infective power. Wheat flour, oatmeal and rice flour were the substances tested, and of these it was discovered that rice flour afforded the best medium. The B. typhosus, staphylococcus aureus and B. pyocyaneous persisted much longer in such media than did the B. diphtheriæ or cholera vibrio, but on the whole it would appear to be unlikely that these food products would prove a source of infection to human beings. Another series of experiments by the same observer demonstrated the fact that whilst preserved foods, such as black pudding, sausages, pies, potted ham, condensed milk, etc., contained relatively numerous microbes belonging to various species, both anærobic and ærobic, they did not contain any organisms that had pathogenic action on rodents. Klein[6] has conducted an investigation to determine whether mussels and cockles are capable of disseminating disease in the same way as oysters. He shows that both these shellfish, if kept for 25 hours in cholera-infected water, retain within their shells the cholera vibrios, and if at the end of that time they are transferred to clean sea-water the vibrios may still be found alive at the end of 24 hours. The same statements hold true in the case of typhoid bacilli. Although mussels and cockles are sometimes eaten raw it is more common to have them cooked. A second series of experiments was therefore undertaken with a view to testing whether cooking would insure the death of these pathogenic bacilli if present, and it was shown that mere immersion in boiling water was insufficient to ensure that end, but that it would probably be secured by prolonged boiling. Gordon[7] reviews the literature relating to the bacteriology of epidemic diarrhœa as communicated by food. He quotes Escherich's remark that the meconium of newly-born infants is sterile, but that by the second day B. lactis ærogenes and B. coli commune abound. Later other organisms are added with the food, and there has been much controversy as to which of these is the determining cause of the diarrhœa. Booker isolated 30 different varieties but thought that the two playing the most prominent part in exciting the disease were streptococci and Proteus vulgaris. The theory of streptococcus causation was also favoured by Holst, Escherich, and Hirsch. These organisms produce injurious changes in the food and bowel contents, and so set up irritation. Cumston attributed infantile diarrhœa to a peculiarly virulent form of B. coli, and Lesage confirmed this and stated that the patient's blood during the attack had agglutinating properties for certain forms of B. coli cultures. Flügge and Lübbert described an ærobic spore-bearing bacillus of the subtilis type, and Klein attributes the disease to invasion by B. enteritidis sporogenes. Outbreaks of diarrhœa amongst adults after eating such articles as pork-pie and veal-pie are undoubtedly of bacterial origin. Klein found in some cases a virulent form of B. coli, in others B. enteritidis sporogenes, and occasionally a vibrio. Tonarelli, Washbourn and Pakes, and Andrews have attributed outbreaks of adult epidemic diarrhœa to streptococcal invasion. Durham investigated 12 epidemics due to poisoning by meat, chiefly beef, and concludes that the infecting organism is the B. enteritidis of Gärtner.

[1] Ann. de l'Instit. Past., June 25, 1902. [2] Ibid. [3] Ann. Rep. Shanghai Munic. Council, 1902. [4] Jour. Med. Research, April 1902. [5] Rep. Med. Officer L. G. B., 1902. [6] Ibid. [7] Practitioner, Aug. 1902.

